# Network analysis of social isolation, loneliness, depression and anxiety among post-discharge rehabilitation patients

**DOI:** 10.3389/fpsyt.2026.1889828

**Published:** 2026-08-19

**Authors:** Yonglan Li, Dongsheng Wang, Shuangting Xu, Jing Xu

**Affiliations:** 1 Mianyang Hospital Affiliated with Chengdu University of Traditional Chinese Medicine, Mianyang Hospital of TCM, Mianyang, China; 2 Sichuan College of Traditional Chinese Medicine, Mianyang, China

**Keywords:** anxiety, depression, loneliness, network analysis, rehabilitation, social isolation

## Abstract

**Background:**

Patients undergoing inpatient or outpatient rehabilitation frequently experience substantial changes in physical functioning, social roles, and daily routines, which may increase vulnerability to social disconnection and psychological distress. This study applied network analysis to explore the interrelationships among social isolation, loneliness, anxiety, and depression in post-discharge rehabilitation patients.

**Aims:**

This study aimed to characterize two network models of social isolation, loneliness, depressive symptoms, and anxiety symptoms among post-discharge rehabilitation patients.

**Methods:**

A cross-sectional study was conducted among patients discharged from the rehabilitation department of a tertiary hospital in China during outpatient follow-up between August 2024 and June 2025. Data on social isolation, loneliness, depressive symptoms and anxiety symptoms were collected from participants. Network structures were estimated using a Graphical Gaussian Model and a Directed Acyclic Graph.

**Results:**

A total of 340 patients discharged from the rehabilitation department were included, with a mean age of 62.1 years. The Graphical Gaussian Model identified a moderately dense network with strong connections between social isolation and loneliness, as well as between several anxiety and depressive symptoms. Fear of awful events, anhedonia, and fatigue emerged as the most central symptoms within the network. The Directed Acyclic Graph analysis suggested that fatigue and fear of awful events functioned as upstream symptoms influencing other emotional and social symptoms.

**Conclusions:**

Among post-discharge rehabilitation patients, social isolation and loneliness in are embedded within a complex network of anxiety and depressive symptoms. The identified central and bridge symptoms may serve as hypothesis-generating candidates for future psychosocial intervention studies. Network analysis provides a useful framework for examining psychological distress in rehabilitation settings.

## Introduction

In recent years, alongside China’s sustained socioeconomic development and marked shifts in population structure and disease patterns, the need for rehabilitation services has become increasingly urgent. Early, standardized rehabilitation delivered during the course of disease can prevent or mitigate functional impairments, improve independence in activities of daily living and quality of life, and reduce the burden on families and society (1, 2). Evidence supports the effectiveness of rehabilitation across diverse conditions. For example, pulmonary rehabilitation improves health status and quality of life in patients with chronic obstructive pulmonary disease and is associated with reduced healthcare utilization and hospitalization rates (3, 4). Rehabilitation is also essential for minimizing disability in patients with severe neurological conditions; combined pharmacological therapy and structured rehabilitation training during recovery can promote restoration of neurological and motor functions and maximize functional outcomes. Moreover, cardiac rehabilitation is a key component of cardiovascular risk reduction and an effective approach to improving long-term health outcomes (5, 6).

Patients undergoing inpatient or outpatient rehabilitation often experience profound changes in physical function, social roles, and daily routines (7, 8). Prolonged hospitalization, mobility limitations, and reduced participation in social activities may substantially restrict patients’ opportunities for social interaction (9, 10). Accumulating evidence indicates that individuals in rehabilitation settings are particularly vulnerable to psychological distress, including social isolation (11), loneliness (12), anxiety and depressive symptoms (13, 14), which may adversely affect functional recovery, treatment adherence, and quality of life.

Social isolation and loneliness, although closely related, represent distinct constructs. Social isolation is defined as an objective state characterized by insufficient quantity and quality of social relationships at the individual, group, community, and broader societal levels (15), whereas loneliness reflects a subjective experience arising when the perceived quantity and/or quality of interpersonal relationships fails to meet an individual’s social needs (16). Social isolation has been linked to a range of adverse health outcomes, including cognitive impairment (17), dysregulation of cardiovascular and neuroendocrine systems (18), disturbed mental health, (19) and an increased risk of mortality (20). Evidence further indicates that social isolation is associated with elevated mortality among patients undergoing stroke rehabilitation (21). Similarly, a cohort study reported that middle-aged and older adults with fluctuating or persistently high levels of social isolation had a higher risk of developing subsequent cardiovascular disease compared with those without such exposure (22). Loneliness has also been shown to exert detrimental effects on health. Studies demonstrate that loneliness predicts stress-related physiological responses, such as elevated blood pressure and increased cortisol levels, as well as maladaptive cognitive and behavioral patterns (23, 24). Moreover, loneliness is associated with an increased risk of morbidity and mortality (20, 25). Importantly, individuals may experience loneliness even in the presence of social contacts, highlighting the complex psychological processes underlying social experiences. In rehabilitation settings, functional impairments and increased dependence on caregivers may exacerbate both objective social isolation and subjective feelings of loneliness. These social and psychological challenges have been associated with heightened emotional distress and poorer health outcomes, highlighting the importance of addressing social isolation and loneliness during the rehabilitation process.

Anxiety and depression are among the most prevalent psychological symptoms observed in rehabilitation populations. Stroke survivors often experience significant psychological difficulties including both depression and anxiety (26). Anxiety and depression have been shown to act as barriers to treatment adherence and life-style changes which may reduce the effect of cardiac rehabilitation (27, 28). Rather than occurring in isolation, these symptoms frequently co-occur and interact with social and emotional factors, such as loneliness and perceived social support. Traditional variable-centered approaches typically conceptualize anxiety and depression as latent constructs or outcomes, which may obscure the dynamic interplay among individual symptoms and social experiences.

A growing body of research suggests that psychological distress may be better understood as a complex system of mutually reinforcing symptoms, where certain symptoms play a central or bridging role in maintaining the overall symptom network. Network analysis has emerged as a powerful methodological framework for modeling psychological phenomena as systems of interacting components (29). By estimating direct associations between individual symptoms while controlling for all others, network analysis enables the identification of central symptoms that may exert a disproportionate influence on the network, as well as bridge symptoms that link different symptom clusters (30).

Applying network analysis to social isolation, loneliness, anxiety, and depression may provide novel insights into how social and emotional symptoms interact within rehabilitation populations and help identify potential targets for more precise and efficient interventions. Although previous studies have documented associations between social isolation, loneliness, and psychological distress, most have relied on total scores or regression-based approaches. Little is known about the symptom-level interaction patterns linking social and emotional experiences among patients in rehabilitation settings. In particular, it remains unclear which specific symptoms serve as central or bridging elements connecting social disconnection with anxiety and depressive symptoms.

Therefore, the present study aimed to explore the network structure of social isolation, loneliness, anxiety, and depression among patients discharged from the rehabilitation department. Specifically, we sought to (1) characterize the overall symptom network, (2) identify central symptoms within the network, and (3) examine bridge symptoms linking social isolation and loneliness with anxiety and depressive symptoms. By elucidating these interaction patterns, this study may provide a theoretical basis for developing targeted psychosocial interventions in rehabilitation care.

## Methods

### Design and participants

A cross-sectional survey was conducted among patients discharged from the Department of Rehabilitation at Mianyang Hospital Affiliated with Chengdu University of Traditional Chinese Medicine. Participants were recruited during outpatient follow-up between August 2024 and June 2025. All participants had completed inpatient rehabilitation and were not receiving inpatient rehabilitation at the time of questionnaire completion. The inclusion criteria were as follows: (1) adults aged ≥18 years who had been discharged from the Department of Rehabilitation and attended outpatient follow-up; (2) absence of cognitive impairment and ability to understand and complete the questionnaires; and (3) provision of informed consent and voluntary participation. The exclusion criteria were: (1) communication disorders or diagnosed mental illness; and (2) malignant tumors or other severe physical illnesses. This study was approved by the Ethics Review Committee of the Mianyang Hospital Affiliated to Chengdu University of Traditional Chinese Medicine. Data were collected using paper-based questionnaires. Participants generally completed the questionnaires independently after receiving standardized instructions from trained researchers. When assistance was required because of reading or writing difficulties, a trained researcher read the items aloud verbatim and recorded the participant’s responses without interpreting the items or influencing the responses. The primary statistical analysis method for data used in this study was network analysis. Considering the sensitivity, specificity, and edge weight correlation of the network, previous studies have indicated that when the sample size is ≥ 250, (31) network analysis can achieve high sensitivity and edge weight correlation, along with moderate specificity. Taking into account a 10% invalid questionnaire rate, the required sample size was determined to be at least 277 participants. A total of 354 participants returned the questionnaires. During data entry, 14 questionnaires were identified as incomplete and were excluded before the analytic dataset was established, corresponding to an incomplete-questionnaire rate of 4.0%. The remaining 340 participants had complete data for all variables included in the analysis and were included in the complete-case analysis. No data were imputed. Because the item-level missing information and demographic characteristics of the 14 excluded participants were not retained, variable-specific missingness and the missing-data mechanism could not be formally assessed.

### Measures

A self-administered structured questionnaire was used to collect sociodemographic and clinically relevant information and to assess social isolation, loneliness, depression, and anxiety, The original study was primarily designed to investigate psychosocial symptom relationships. Detailed rehabilitation-related characteristics, including rehabilitation duration, rehabilitation intensity, objective functional status, mobility level, disease severity, and time since diagnosis or injury, were not systematically collected and therefore could not be included in the present analysis.

### Socio-demographic and clinical variables

The demographic and clinical questionnaire collected information on participants’ age, gender, marital status, educational level, monthly income, place of residence, live status, employment status, and classification of diseases.

### Social isolation

The Social Isolation Scale (SIS) was developed by Nicholson et al. (32). The scale comprises two dimensions: objective connectedness and subjective belongingness. The connectedness items (items 1-3) ask respondents to report the frequency of contact with family, friends, and neighbors on a five-point scale ranging from 0 to 6 or more contacts. The belongingness items (items 4–6) ask respondents to indicate their agreement with statements about relationships and activities, from strongly disagree to strongly agree. Item 5 is reverse-scored. Total scores range from 0 to 24, with lower scores indicating greater social isolation. A score of ≤15 indicates a risk of social isolation. In this study, Cronbach’s alpha values for connectedness and belongingness were 0.810 and 0.819, respectively.

### Loneliness

The six-item University of California, Los Angeles Loneliness Scale, Short Form (ULS-6) (33) was used to assess loneliness. Items are rated on a four-point Likert scale ranging from 1 (never) to 4 (often). Total scores range from 6 to 24, with higher scores indicating greater loneliness. A score of ≥10 indicates loneliness. In this study, Cronbach’s alpha for the ULS-6 was 0.911.

### Depression

The Patient Health Questionnaire-9 (PHQ-9) (34) comprises of nine items that assess depressive symptoms during the preceding two weeks. Each item is scored from 0 (not at all) to 3 (nearly every day), yielding a total score of 0-27. Higher scores indicate a greater depressive symptom. severity. In this study, Cronbach’s alpha for the PHQ-9 was 0.889.

### Anxiety

The Generalized Anxiety Disorder-7 (GAD-7) (35) was used to assess anxiety symptoms during the preceding two weeks. The scale comprises seven items, each scored from 0 (not at all) to 3 (nearly every day), yielding a total score of 0-21. Higher scores are indicate greater anxiety severity. In this study, Cronbach’s alpha for the GAD-7 was 0.876.

### Statistical analysis

Statistical analyses were performed using SPSS 27.0 and R 4.5.1 software. Questionnaires with incomplete responses were excluded before data entry, and a complete-case analysis was performed. No imputation was used because the final analytic dataset contained no missing values. Because incomplete questionnaires had been discarded during data entry, item-level missingness and the missing-data mechanism could not be retrospectively evaluated. Descriptive analysis was performed using the SPSS 27.0, employing frequencies (percentages) for qualitative variables and means with standard deviations for quantitative variables. To explore multiple associations among the two social isolation components, loneliness, nine depressive symptoms, and seven anxiety symptoms, we conducted graphical gaussian model (GGM) and directed acyclic graph (DAG) network analyses using R version 4.5.1 along with various R packages. Before network estimation, potential node redundancy was assessed using the goldbricker function in the R package networktools. This procedure evaluates whether pairs of nodes exhibit highly similar correlation patterns with all other nodes in the network. Using the default criteria, node pairs with a zero-order correlation of at least 0.50 and fewer than 25% significantly different correlations with the remaining nodes were considered potentially redundant. Communities were specified *a priori* based on the theoretical structure and scoring framework of the corresponding instruments rather than identified using a data-driven community-detection algorithm. The social isolation total-score node was assigned to the social isolation community, the loneliness total-score node to the loneliness community, the nine PHQ-9 symptom nodes to the depression community, and the seven GAD-7 symptom nodes to the anxiety community.

In the undirected network structure, variables are depicted as nodes, while their connection between two variables is referred to as edges. The thickness of each edge indicates the strength of these associations, with thicker edges signifying stronger relationships. The graphical least absolute shrinkage and selection operator (gLASSO), combined with the extend Bayesian information criterion (EBIC) was employed to estimate the network (36). The EBIC tuning hyperparameter γ was set to 0.50. In the network analysis, two centrality indices, expected influence (37) (EI) (i.e., a given node’ s cumulative influence in the network, with a higher EI indicating a more important node), bridge expected influence (30) (BEI) (i.e., the cumulative influence of a node from one cluster on all nodes of another cluster), must be computed to explore the importance of individual variables within the network. We estimated the predictability of nodes, which denotes to what extent a node’s variance can be predicted by the variances of the nodes connected to it. The Bootstrap method was employed to compute 95% confidence intervals (CIs) that assess the accuracy of edge weights, and estimate the correlation stability coefficients (CS-Cs). Epskamp et al. (29) recommend to not interpret CS-coefficients lower than 0.25, but preferably only interpret those larger than 0.50.

A DAG is a dynamic Bayesian network approach that can gives us information about both the strength and the direction of connections between multiple variables from cross-sectional data (38). A DAG including all variables was estimated using the hill-climbing structure-learning algorithm implemented in the R package bnlearn (39). During structure learning, edges were added, removed, or reversed to identify the network structure with the optimal Bayesian information criterion (BIC) score. To assess the stability of the estimated network, nonparametric bootstrapping with 10,000 resamples was performed, and a network was estimated for each bootstrap sample (40). For each possible edge, bootstrap strength was calculated as the proportion of bootstrap networks in which the edge was present. Edges were retained using the data-driven optimal cut-point proposed by Scutari and Nagarajan (41), which was designed to balance sensitivity and specificity. For each retained edge, directional probability was calculated as the proportion of bootstrap networks containing that edge in which it was oriented from symptom X to symptom Y. The X-to-Y orientation was depicted in the final DAG when its directional probability was ≥0.51. This threshold was selected as a simple majority rule, because a value of 0.50 indicates equal support for the two possible orientations. The threshold was used only to identify the more frequently supported direction across bootstrap samples and should not be interpreted as evidence of temporal precedence or causality.

## Results

### Sample characteristics

Of the 354 returned questionnaires, 14 (4.0%) were excluded because of incomplete responses, leaving 340 complete questionnaires for the final analysis. Among the 340 patients, the mean age was 62.1 (SD = 9.55) years. The majority of participants were female, 85.6% were married, 27.6% had bachelor or above degree,54.4% had a better monthly income, and 75.9% lived in rural area. Other characteristics of the patients are summarized in **Table 1**. Outcome of descriptive analysis of social isolation, loneliness, depression and anxiety are shown in **Table 2**. Of the 340 participants, 41 (12.10%) had clinically significant depressive symptoms, defined as a PHQ-9 total score ≥10, and 21 (6.20%) had clinically significant anxiety symptoms, defined as a GAD-7 total score ≥10. In addition, Among the 340 participants, 161 (47.4%) were identified as being at risk of social isolation, and 121 (35.6%) met the cutoff criterion for loneliness.

**Table 1:** Demographic characteristics of post-discharge rehabilitation patients (N = 340).

Variables	N/(M ± SD)	Percentage (%)
Age (years)	62.10 ± 9.55	
Gender		
Male	153	45
Female	187	55
Marital status		
Married	291	85.6
Divorced/widowed	49	14.4
Educational level		
Primary school or below	101	29.7
Middle school	77	22.6
High school	68	20
Bachelor or above	94	27.6
Monthly income (¥)		
< 3000	40	11.8
3000~6000	115	33.8
> 6000	185	54.4
Place of residence		
Urban area	82	24.1
Rural area	258	75.9
Live status		
Alone	53	15.6
Live with others	287	84.4
Employment status		
Workers	139	40.9
Retired	125	36.8
Unemployed	76	22.4
Classification of diseases		
Low back pain	87	25.6
Cervical spondylosis	75	22.1
Osteoarthritis	87	25.6
Osteoporosis	43	12.6
Stroke	7	2.1
Shingles	18	5.3
Bell’s palsy	23	6.8

**Table 2:** Descriptive analysis of SI, LO, PHQ and GAD items(N = 340).

Number	Item	M	SD
SI	Social Isolation	15.71	4.92
LO	Loneliness	9.79	4.60
PHQ	Depressive symptom total score	3.94	4.80
PHQ1	Anhedonia	0.57	0.81
PHQ2	Depressed Mood	0.58	0.77
PHQ3	Sleep Disturbance	0.56	0.76
PHQ4	Fatigue	0.59	0.83
PHQ5	Appetite Disturbance	0.36	0.62
PHQ6	Feelings of Worthlessness	0.42	0.71
PHQ7	Concentration Difficulties	0.35	0.64
PHQ8	Psychomotor Agitation/Retardation	0.30	0.64
PHQ9	Suicide Ideation	0.23	0.51
GAD	Anxiety symptom total score	2.68	3.70
GAD1	Nervous	0.45	0.67
GAD2	Uncontrollable Worry	0.47	0.69
GAD3	Worry about Many Things	0.49	0.70
GAD4	Unable to Relax	0.36	0.64
GAD5	Restlessness	0.28	0.56
GAD6	Easily Annoyed	0.41	0.67
GAD7	Fear of Awful Events	0.23	0.52

### Graphical Gaussian model network

**Figure 1:**
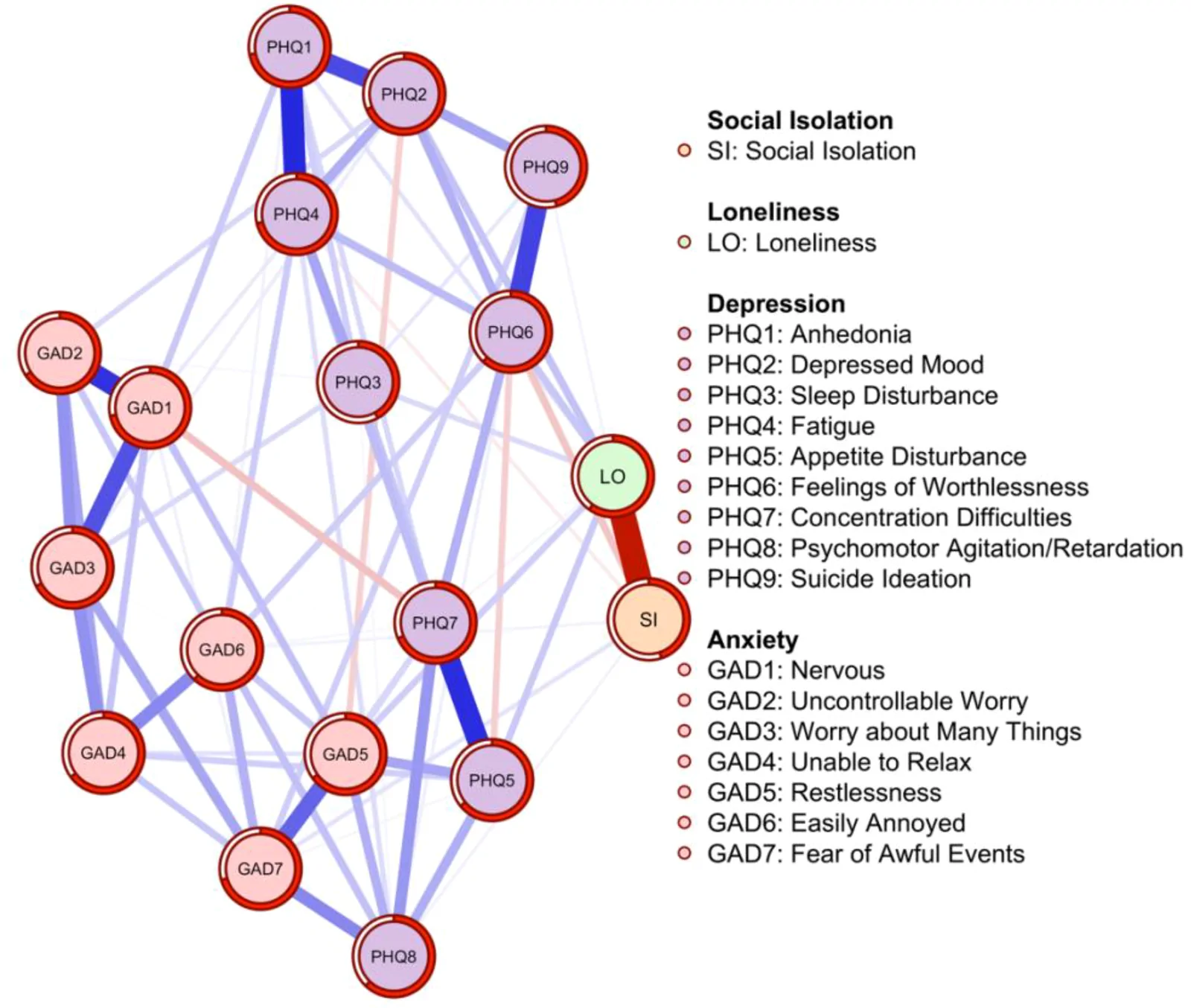
Undirected network of social isolation, loneliness, depression and anxiety among post-discharge rehabilitation patients. Blue lines represent positive correlations, and red lines represent negative correlations. The red rings around the nodes indicate the predictability of this node.

**Figure 2:**
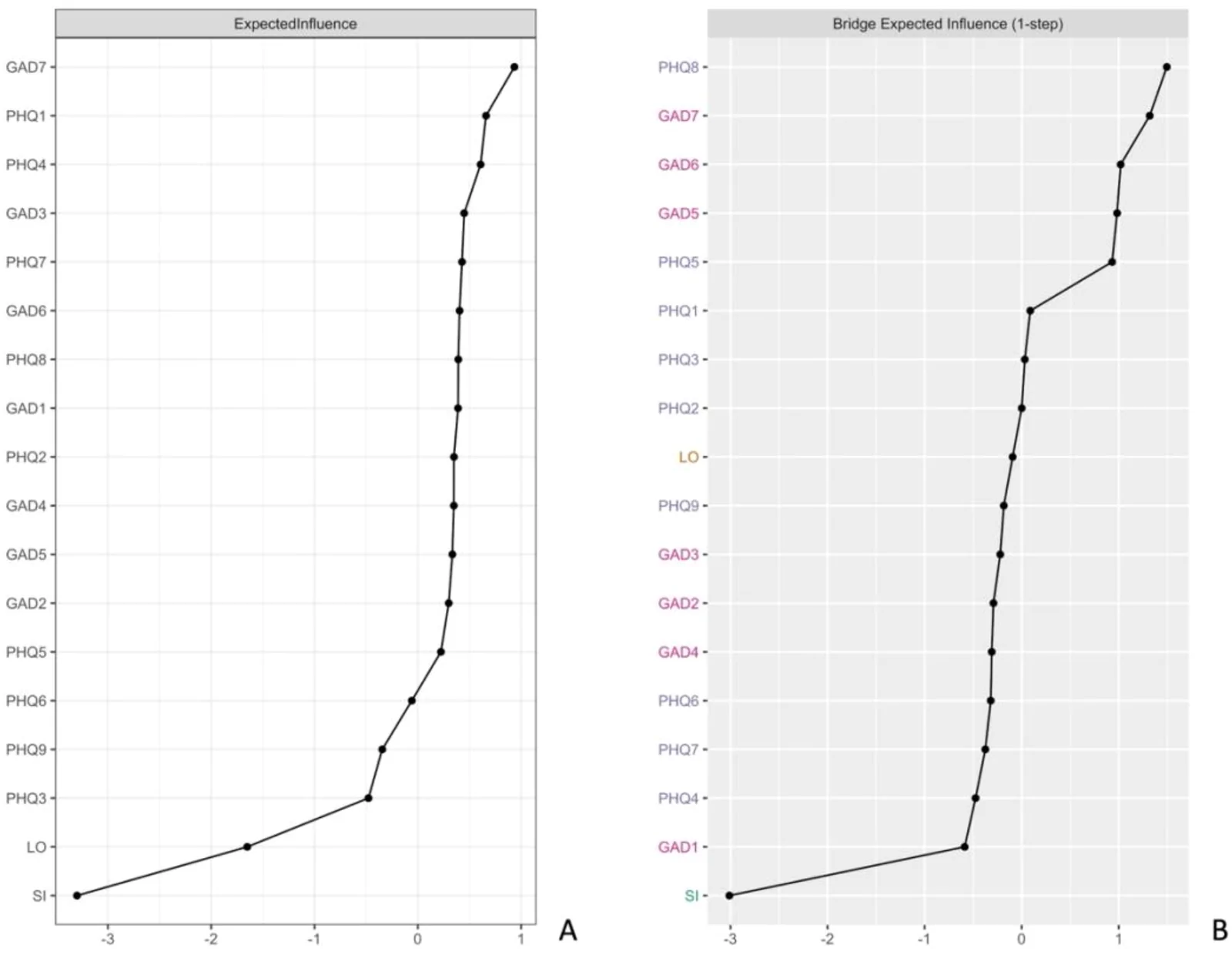
Expected influence **(A)** and bridge expected influence **(B)** in the undirected network. SI, Social Isolation; LO, loneliness; PHQ1, Anhedonia; PHQ2, Depressed Mood; PHQ3, Sleep Disturbance; PHQ4, Fatigue; PHQ5, Appetite Disturbance; PHQ6, Feelings of Worthlessness; PHQ7, Concentration Difficulties; PHQ8, Psychomotor Agitation/Retardation; PHQ9, Suicide Ideation; GAD1, Nervous; GAD2, Uncontrollable Worry; GAD3, Worry about Many Things; GAD4, Unable to Relax; GAD5, Restlessness; GAD6, Easily Annoyed; GAD7, Fear of Awful Events.

The node redundancy analysis did not identify any potentially redundant pairs among the social isolation, loneliness, PHQ-9, and GAD-7 nodes. Therefore, all original nodes were retained in the network analysis. **Figure 1** depicts the network of variables as estimated with the GGM, which illustrates the regularized partial correlations between variables. The density of the network is represented by the proportion of non-zero edges. Of 171 possible edges, 83 (48.5%) edges were evident in the final network, with 73 positive and 10 negative connections. The strongest edges in the network include social isolation with loneliness (edge weight = -0.461); anhedonia with fatigue (edge weight = 0.392); appetite disturbance with concentration difficulties (edge weight = 0.378); nervous with uncontrollable (edge weight = 0.367). The detailed corresponding weighted adjacency matrix are provided in **Supplementary Table 1**. The standardized estimate of EI and BEI indices for the undirected network is shown in **Figure 2**. Fear of awful events had the highest expected influence, followed by anhedonia and fatigue, whereas loneliness and social isolation showed relatively low expected influence. Psychomotor agitation/retardation, fear of awful events, and easily annoyed had the highest bridge expected influence values. The mean node predictability was 0.627, ranging from 0.421 to 0.720, indicating that the other nodes in the fitted nodewise models explained, on average, 62.7% of the variance in each node. Anhedonia had the highest predictability (R² = 0.720), whereas sleep disturbance had the lowest predictability (R² = 0.421). Numerical estimates of expected influence, bridge expected influence, and predictability for all nodes are presented in **Supplementary Table 2**.

**Supplementary Figure 1** presents the network stability assessed using the case-dropping bootstrap procedure. The CS-C for EI was 0.75 and BEI was 0.68, indicating high network stability. **Supplementary Figure 2** presents the bootstrapped 95% CIs around the edge weights. Narrower CIs indicated that the network structure was accurate and reliable. Additionally, the bootstrapped difference tests suggested that and the node centrality was statistically different from one another in individual comparisons (**Supplementary Figure 3**), and a considerable proportion of comparisons among edge weights proved to be statistically significant (**Supplementary Figure 4**).

### Directed acyclic graph network

**Figure 3:**
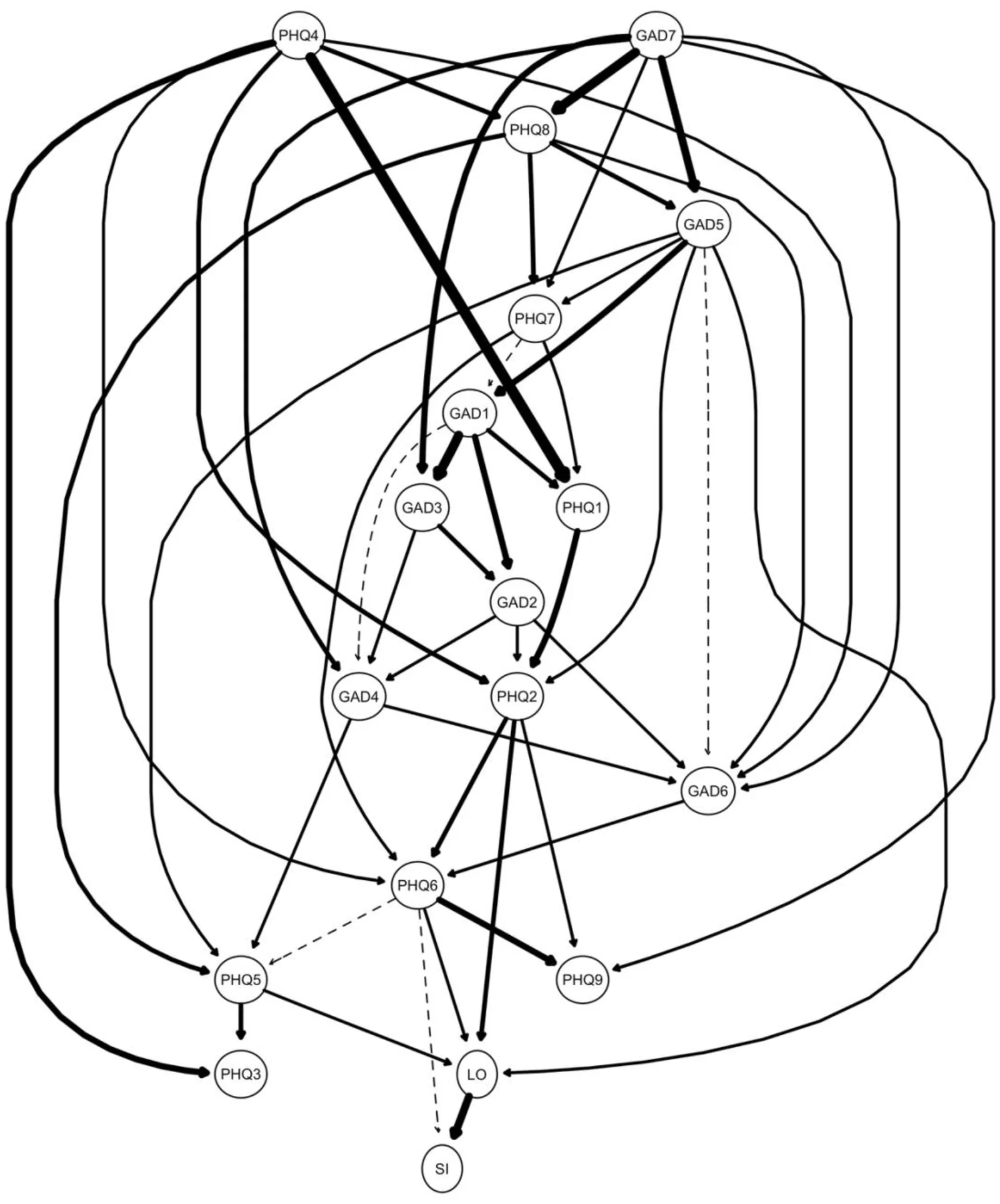
Directed network of social isolation, loneliness, depression and anxiety among post-discharge rehabilitation patients. SI, Social Isolation; LO, loneliness; PHQ1, Anhedonia; PHQ2, Depressed Mood; PHQ3, Sleep Disturbance; PHQ4, Fatigue; PHQ5, Appetite Disturbance; PHQ6, Feelings of Worthlessness; PHQ7, Concentration Difficulties; PHQ8, Psychomotor Agitation/Retardation; PHQ9, Suicide Ideation; GAD1, Nervous; GAD2, Uncontrollable Worry; GAD3, Worry about Many Things; GAD4, Unable to Relax; GAD5, Restlessness; GAD6, Easily Annoyed; GAD7, Fear of Awful Events.

**Figure 3** illustrates the DAG that resulted from the Bayesian network analysis. In the figure, each edge’s thickness is in proportion to its strength, and edges that were identified in less than 50% of the bootstrapped networks are depicted with dotted lines. The edge thickness represents the BIC, meaning the thicker the line, the greater the importance that edge has in the fit of the model. Notably, persistent fatigue and fear of awful events might happen occupied relatively upstream positions in the estimated DAG. These could directly or indirectly trigger other behaviors or symptoms. Further, the findings suggested that expression of sleep disturbance, loneliness, and social isolation were downstream behaviors and seemed to occur because of other behaviors.

## Discussion

In this study, GGM and DAG analyses were used to characterize the network structure of social isolation, loneliness, depressive symptoms, and anxiety symptoms among post-discharge rehabilitation patients and to identify central, bridge, and directionally prominent symptoms. The GGM indicated that psychomotor agitation/retardation, fear that something awful might happen, and becoming easily annoyed or irritable were key bridge symptoms linking social isolation and loneliness with depressive and anxiety symptoms. In the estimated DAG, fatigue and fear that something awful might happen occupied relatively upstream positions and had direct or indirect directed connections with social isolation and loneliness through other depressive and anxiety symptoms.

Although no node pairs met the predefined criteria for redundancy, some conceptual overlap between depressive and anxiety symptoms should still be acknowledged. The PHQ-9 and GAD-7 assess distinct symptom domains but also capture related aspects of general psychological distress. In particular, somatic and arousal-related symptoms, such as concentration difficulties, psychomotor agitation or retardation, difficulty relaxing, and restlessness, may partially reflect shared distress processes. Therefore, strong cross-community edges and bridge centrality should not necessarily be interpreted as indicating entirely distinct symptom-to-symptom relationships, as they may partly reflect overlapping symptom content. Moreover, symptoms such as fatigue, sleep disturbance, concentration difficulties, and psychomotor changes may be influenced by physical illness and rehabilitation burden, further complicating the distinction between depressive, anxiety, and somatic manifestations.

In this study, depressive symptom of psychomotor agitation/retardation, anxiety symptom of fear of awful events and easily annoyed emerged as key bridge nodes linking the social isolation and loneliness cluster with depressive and anxiety symptoms. Our findings suggest that not all depressive or anxiety symptoms are equally involved in this process; instead, a small set of behaviorally and cognitively loaded symptoms appears to play a disproportionate role in connecting social isolation and loneliness with internalizing psychopathology among post-discharge rehabilitation patients.

Psychomotor agitation/retardation reflects observable slowing or restlessness in thinking and movement and has been associated with greater depressive symptom severity and biological aspects of depression (42, 43). Psychomotor slowing may coexist with reduced opportunities for social participation, withdrawal from meaningful roles, and a greater subjective sense of disconnection from others (44, 45). Conversely, loneliness and social isolation may also be associated with reduced motivation, energy, and engagement in rehabilitation activities (44). The bridge position of psychomotor agitation/retardation therefore suggests that behavioral and motor symptoms may be relevant to the association between social disconnection and depressive symptoms. However, the present network cannot determine the direction of these relationships.

Fear that something awful might happen and becoming easily annoyed or irritable may represent cognitive and interpersonal aspects of anxiety that are particularly relevant in rehabilitation settings. Catastrophic expectations may be salient among patients who are concerned about disease progression, functional decline, or dependency on others (46–48). When such worries are experienced in the context of limited social support, they may be associated with increased rumination, heightened vigilance, and avoidance of social situations, as well as greater social isolation and loneliness (49–51). Becoming easily annoyed or irritable may be associated with interpersonal difficulties involving family members, healthcare staff, or other patients, and may coincide with reduced perceived social connectedness (52). The prominent bridge centrality of these two symptoms suggests that a profile characterized by catastrophic worry and irritability may be particularly relevant to the association between anxiety and social disconnection.

Rehabilitation-specific network evidence remains limited and has predominantly focused on single diagnostic populations, particularly stroke and spinal cord injury. In persons with stroke, Ashaie et al. (53) found that depressed mood was consistently central across networks estimated at discharge from inpatient rehabilitation and at 3- and 12-month post-discharge follow-ups, with greater symptom connectivity observed after discharge. A recent study of older stroke survivors similarly identified feeling depressed, feeling nervous or fearful, and loneliness as prominent symptoms within the depressive symptom network (54). These findings support the relevance of affective distress and social disconnection during post-discharge recovery. In patients with spinal cord injury, however, concentration difficulties and trouble relaxing showed high centrality, whereas nervousness and feelings of failure occupied prominent bridging positions between psychological and social-adjustment domains (55). This differs from the present findings, in which psychomotor agitation/retardation, fear of awful events, and becoming easily annoyed or irritable occupied prominent bridge positions. Such variation may reflect differences in diagnosis, functional impairment, measurement instruments, rehabilitation stage, and social context. Overall, rehabilitation-specific network studies remain sparse, and, to our knowledge, few studies have simultaneously examined social isolation, loneliness, depressive symptoms, and anxiety symptoms in a heterogeneous post-discharge rehabilitation population. The present findings therefore extend this emerging literature but require replication in diagnosis-specific and longitudinal rehabilitation samples.

Taken together, these findings are consistent with the symptom-level network perspective, which suggests that individual symptoms may differ in their structural roles across psychological domains (56). The identification of psychomotor agitation/retardation, fear that something awful might happen, and becoming easily annoyed or irritable as bridge symptoms may help generate hypotheses regarding symptoms that warrant closer attention among post-discharge rehabilitation patients experiencing both social disconnection and emotional distress. These symptoms could be considered as part of a comprehensive psychosocial assessment; however, the present study did not evaluate their screening accuracy or predictive performance. Moreover, bridge centrality reflects the structural position of a symptom within the estimated network and does not demonstrate that targeting the symptom would improve other symptoms or clinical outcomes. Behavioral activation, structured activity scheduling, psychoeducation, cognitive-behavioral techniques, and emotion-regulation strategies may represent candidate approaches for future intervention research. Their effectiveness in reducing social isolation, loneliness, depressive symptoms, or anxiety symptoms should be evaluated in prospective longitudinal and randomized intervention studies.

In addition to the undirected GGM, the estimated DAG placed fatigue and fear that something awful might happen in relatively upstream positions. These symptoms had directed connections with several depressive and anxiety symptoms and were also directly or indirectly connected with social isolation and loneliness. In the bootstrap analysis, these orientations were more frequently supported than their reverse orientations. However, the upstream positions represent relative structural locations within the statistically estimated DAG and do not demonstrate that fatigue or fear that something awful might happen temporally preceded or causally influenced the other symptoms.

Fatigue is a common and clinically burdensome symptom in rehabilitation settings and may reflect the combined effects of physical illness, treatment, functional impairment, physical deconditioning, and emotional distress (57, 58). It may coexist with reduced participation in rehabilitation exercises, daily activities, social roles, and interpersonal interactions (59, 60). In the estimated DAG, fatigue occupied a relatively upstream position and had directed connections with several depressive and anxiety symptoms. However, this finding should not be interpreted as indicating that fatigue represents a purely depressive process or initiates a causal sequence. Among post-discharge rehabilitation patients, fatigue may arise directly from pain, disease severity, medication effects, sleep disruption, functional limitations, or rehabilitation burden. Psychomotor agitation/retardation, fear that something awful might happen, and feeling easily annoyed emerged as key bridge nodes linking the social isolation and loneliness communities with depressive and anxiety symptoms. These findings suggest that individual depressive and anxiety symptoms differ in their structural roles; a small set of behavioral and cognitive symptoms may be particularly relevant to the association between social disconnection and internalizing psychopathology among post-discharge rehabilitation patients.

Fear that something awful might happen represents a cognitive component of anticipatory anxiety and catastrophic thinking. Post-discharge rehabilitation patients may experience concerns about disease progression, future disability, and dependency on others (61). Such concerns may coexist with hypervigilance, somatic tension, irritability, and social avoidance (62, 63). In the estimated DAG, fear that something awful might happen had direct and indirect directed connections with social isolation and loneliness through other anxiety and depressive symptoms. This pattern indicates that the symptom occupied a structurally prominent position, but it does not demonstrate that it initiated subsequent emotional or behavioral changes. Its identification as both a bridge symptom in the GGM and an upstream-positioned node in the DAG suggests that it warrants further investigation in longitudinal studies.

From a clinical perspective, the upstream positions of fatigue and fear that something awful might happen in the estimated DAG generate the hypothesis that these symptoms may warrant further assessment among post-discharge rehabilitation patients with co-occurring social and emotional difficulties. Future longitudinal studies are needed to determine whether changes in these symptoms temporally precede or predict changes in depression, anxiety, social isolation, or loneliness. Potential approaches, including graded activity, behavioral activation, psychoeducation, and cognitive-behavioral strategies, could be evaluated in future intervention studies (64, 65). However, because no intervention was administered in the present study, our findings do not demonstrate that targeting fatigue or catastrophic worry would prevent, reduce, or disrupt social isolation, loneliness, or other emotional symptoms. Therefore, symptom-specific clinical recommendations should await confirmation from longitudinal and randomized intervention studies.

### Limitations

Several limitations should be acknowledged. First, the use of a single-center convenience sample may have introduced selection bias and limited the generalizability of the findings. The sample was also diagnostically heterogeneous and was dominated by musculoskeletal conditions, whereas relatively few participants had stroke, Bell’s palsy, or shingles. Although these diagnoses were pooled to examine transdiagnostic symptom relationships, differences in disease severity, functional impairment, pain, prognosis, and social participation may have influenced the estimated network. The resulting network may therefore represent an averaged structure that obscures diagnosis-specific associations. Furthermore, the small and imbalanced diagnostic subgroups precluded reliable subgroup network comparisons.

Second, the psychosocial variables were assessed using self-report questionnaires and may therefore have been affected by recall, response, and common-method biases. Symptoms such as fatigue, sleep disturbance, concentration difficulties, and psychomotor changes may reflect both depressive psychopathology and physical illness or rehabilitation-related burden. Because objective measures of functional status, disease severity, pain, medication use, and rehabilitation exposure were unavailable, we could not distinguish adequately between psychological and somatic contributions to these symptoms.

Third, demographic, clinical, and rehabilitation-related variables—including age, sex, diagnosis, disease duration, and rehabilitation stage—were not incorporated into the network model. Residual confounding therefore cannot be excluded, and the observed edges should not be interpreted as fully adjusted symptom-to-symptom relationships.

Fourth, the cross-sectional design and absence of longitudinal follow-up preclude temporal and causal inference. The orientations identified in the DAG represent statistically supported conditional dependencies under specific modeling assumptions; they do not demonstrate that upstream-positioned symptoms temporally precede or cause other symptoms. Moreover, because the study did not assess intervention effects, predictive performance, or screening accuracy, the identified central, bridge, and upstream-positioned symptoms should be regarded as hypothesis-generating candidates rather than validated clinical targets.

Finally, 14 incomplete questionnaires were excluded before data entry, and detailed information about the missing items and excluded participants was not retained. Although the excluded proportion was relatively low (4.0%), the missing-data mechanism could not be evaluated, and selection bias resulting from the analysis of complete questionnaires cannot be ruled out.

Future multicenter longitudinal studies should include larger diagnosis-specific samples, comprehensive demographic and clinical covariates, objective measures of functional status and rehabilitation exposure, and systematic documentation of missing data. Such studies are needed to assess the temporal stability, diagnosis specificity, and potential clinical relevance of the observed network structure.

## Conclusion

In summary, social isolation and loneliness among post-discharge rehabilitation patients were connected to specific depressive and anxiety symptoms within the estimated networks. The GGM identified psychomotor agitation/retardation, fear of awful events, and feeling easily annoyed as prominent bridge nodes, whereas the DAG placed fatigue and fear of awful events in relatively upstream positions with direct or indirect directed paths to social isolation and loneliness. These findings are hypothesis-generating and suggest symptoms that warrant closer assessment and prospective study. Because the networks were estimated from cross-sectional observational self-report data, the inferred orientations must be interpreted cautiously and confirmed in longitudinal and intervention studies before clinical targeting is recommended.

## Data Availability

The original contributions presented in the study are included in the article/**Supplementary Material**, further inquiries can be directed to the corresponding author/s.
