# Supplementary material for: Network analysis of social isolation, loneliness, depression and anxiety among post-discharge rehabilitation patients
Source: Front Psychiatry. 2026 Aug 19;17:1889828. doi: 10.3389/fpsyt.2026.1889828 (PMC13534128; doi:10.3389/fpsyt.2026.1889828)
Supplement: Supplementary file 1 [file Supplementaryfile1.docx]

**Supplementary Table** [**1**](https://link.springer.com/article/10.1186/s12888-023-04971-x#MOESM1). Weighted adjacency matrix.

|  | **SI** | **LO** | **PHQ1** | **PHQ2** | **PHQ3** | **PHQ4** | **PHQ5** | **PHQ6** | **PHQ7** | **PHQ8** | **PHQ9** | **GAD1** | **GAD2** | **GAD3** | **GAD4** | **GAD5** | **GAD6** | **GAD7** |
| --- | --- | --- | --- | --- | --- | --- | --- | --- | --- | --- | --- | --- | --- | --- | --- | --- | --- | --- |
| **SI** | 0 |  |  |  |  |  |  |  |  |  |  |  |  |  |  |  |  |  |
| **LO** | -0.461 | 0 |  |  |  |  |  |  |  |  |  |  |  |  |  |  |  |  |
| **PHQ1** | -0.006 | 0.059 | 0 |  |  |  |  |  |  |  |  |  |  |  |  |  |  |  |
| **PHQ2** | -0.02 | 0.113 | 0.325 | 0 |  |  |  |  |  |  |  |  |  |  |  |  |  |  |
| **PHQ3** | 0 | 0.073 | 0.092 | 0.012 | 0 |  |  |  |  |  |  |  |  |  |  |  |  |  |
| **PHQ4** | -0.044 | 0 | 0.392 | 0.149 | 0.163 | 0 |  |  |  |  |  |  |  |  |  |  |  |  |
| **PHQ5** | 0 | 0.101 | 0 | -0.005 | 0.111 | -0.017 | 0 |  |  |  |  |  |  |  |  |  |  |  |
| **PHQ6** | -0.109 | 0.105 | 0 | 0.144 | 0 | 0.127 | -0.091 | 0 |  |  |  |  |  |  |  |  |  |  |
| **PHQ7** | 0 | 0.024 | 0.075 | 0 | 0 | 0.122 | 0.378 | 0.143 | 0 |  |  |  |  |  |  |  |  |  |
| **PHQ8** | 0.029 | 0 | 0.008 | 0 | 0.02 | 0.065 | 0.149 | 0.034 | 0.188 | 0 |  |  |  |  |  |  |  |  |
| **PHQ9** | 0 | 0.027 | 0 | 0.163 | 0.047 | 0 | 0 | 0.339 | 0 | 0 | 0 |  |  |  |  |  |  |  |
| **GAD1** | 0 | 0 | 0.102 | 0.044 | 0 | 0 | 0 | 0 | -0.117 | 0 | 0 | 0 |  |  |  |  |  |  |
| **GAD2** | 0.002 | 0 | 0 | 0.076 | 0.018 | 0 | 0 | 0 | 0 | 0 | 0 | 0.367 | 0 |  |  |  |  |  |
| **GAD3** | 0 | 0 | 0 | 0.04 | 0.073 | 0 | 0 | 0 | 0 | 0 | 0 | 0.311 | 0.195 | 0 |  |  |  |  |
| **GAD4** | 0 | 0 | 0 | 0 | 0 | 0 | 0.09 | 0 | 0 | 0 | 0.002 | 0.112 | 0.169 | 0.201 | 0 |  |  |  |
| **GAD5** | 0 | 0.093 | 0 | -0.089 | 0 | 0 | 0.162 | 0 | 0.076 | 0.144 | 0 | 0.116 | 0 | 0 | 0.055 | 0 |  |  |
| **GAD6** | 0.029 | 0.009 | 0.029 | 0 | 0 | 0.098 | 0 | 0.094 | 0.02 | 0.115 | 0 | 0 | 0.103 | 0.011 | 0.213 | 0.106 | 0 |  |
| **GAD7** | 0.055 | 0 | 0 | 0 | 0.007 | 0 | 0.022 | 0 | 0.074 | 0.215 | 0.091 | 0.033 | 0 | 0.16 | 0.107 | 0.279 | 0.143 | 0 |

*Note:* SI: Social Isolation; LO: loneliness; PHQ1: Anhedonia; PHQ2: Depressed Mood; PHQ3: Sleep Disturbance; PHQ4: Fatigue; PHQ5: Appetite Disturbance; PHQ6: Feelings of Worthlessness; PHQ7: Concentration Difficulties; PHQ8: Psychomotor Agitation/Retardation; PHQ9: Suicide Ideation; GAD1: Nervous; GAD2: Uncontrollable Worry; GAD3: Worry about Many Things; GAD4: Unable to Relax; GAD5: Restlessness; GAD6: Easily Annoyed; GAD7: Fear of Awful Events.

**Supplementary Table 2**. Numerical estimates of expected influence, bridge expected influence, and node predictability

| **Node_ID** | **Expected_Influence_Raw** | **Expected_Influence_Z** | **Bridge_Expected_Influence_Raw** | **Bridge_Expected_Influence_Z** | **Predictability_R2** |
| --- | --- | --- | --- | --- | --- |
| **SI** | -0.525 | -3.3 | -0.525 | -3.011 | 0.45 |
| **LO** | 0.141 | -1.651 | 0.141 | -0.093 | 0.599 |
| **PHQ1** | 1.076 | 0.661 | 0.183 | 0.088 | 0.72 |
| **PHQ2** | 0.95 | 0.35 | 0.163 | 0 | 0.688 |
| **PHQ3** | 0.616 | -0.477 | 0.17 | 0.032 | 0.421 |
| **PHQ4** | 1.055 | 0.608 | 0.054 | -0.475 | 0.713 |
| **PHQ5** | 0.899 | 0.224 | 0.375 | 0.931 | 0.641 |
| **PHQ6** | 0.786 | -0.057 | 0.09 | -0.319 | 0.619 |
| **PHQ7** | 0.982 | 0.429 | 0.077 | -0.375 | 0.687 |
| **PHQ8** | 0.967 | 0.393 | 0.504 | 1.495 | 0.622 |
| **PHQ9** | 0.67 | -0.344 | 0.121 | -0.184 | 0.458 |
| **GAD1** | 0.966 | 0.39 | 0.029 | -0.587 | 0.717 |
| **GAD2** | 0.93 | 0.3 | 0.096 | -0.291 | 0.659 |
| **GAD3** | 0.99 | 0.449 | 0.112 | -0.221 | 0.675 |
| **GAD4** | 0.95 | 0.349 | 0.092 | -0.309 | 0.649 |
| **GAD5** | 0.944 | 0.335 | 0.387 | 0.982 | 0.65 |
| **GAD6** | 0.972 | 0.405 | 0.396 | 1.019 | 0.62 |
| **GAD7** | 1.187 | 0.936 | 0.464 | 1.318 | 0.706 |

Note. SI: Social Isolation; LO: loneliness; PHQ1: Anhedonia; PHQ2: Depressed Mood; PHQ3: Sleep Disturbance; PHQ4: Fatigue; PHQ5: Appetite Disturbance; PHQ6: Feelings of Worthlessness; PHQ7: Concentration Difficulties; PHQ8: Psychomotor Agitation/Retardation; PHQ9: Suicide Ideation; GAD1: Nervous; GAD2: Uncontrollable Worry; GAD3: Worry about Many Things; GAD4: Unable to Relax; GAD5: Restlessness; GAD6: Easily Annoyed; GAD7: Fear of Awful Events; EI, expected influence; BEI, bridge expected influence; R², node predictability. Expected influence and bridge expected influence are presented as raw estimates and standardized z scores. Predictability represents the proportion of variance in each node accounted for by the other variables retained in its fitted nodewise model.

**Supplementary Figure 1.** Stability of centrality indices as assessed using the case-dropping bootstrap method.

*Note.* The x-axis represents the percentage of cases of the original sample used at each step. The y-axis represents the average of correlations between the centrality indices from the original network and the centrality indices from the networks that were re-estimated after excluding increasing percentages of cases.

SI: Social Isolation; LO: loneliness; PHQ1: Anhedonia; PHQ2: Depressed Mood; PHQ3: Sleep Disturbance; PHQ4: Fatigue; PHQ5: Appetite Disturbance; PHQ6: Feelings of Worthlessness; PHQ7: Concentration Difficulties; PHQ8: Psychomotor Agitation/Retardation; PHQ9: Suicide Ideation; GAD1: Nervous; GAD2: Uncontrollable Worry; GAD3: Worry about Many Things; GAD4: Unable to Relax; GAD5: Restlessness; GAD6: Easily Annoyed; GAD7: Fear of Awful Events.

**Supplementary Figure 2.** Accuracy of edge weights for the current network model.

*Note.* The gray area represents the 95% Confidence Intervals of edge weights, estimated with the non-parametric bootstrap procedure. Wide intervals indicate lower stability and narrow intervals indicate higher stability. The red dots indicate the sample values, while the black dots indicate the values of each edge weight, ordered from the highest to the lowest value.

SI: Social Isolation; LO: loneliness; PHQ1: Anhedonia; PHQ2: Depressed Mood; PHQ3: Sleep Disturbance; PHQ4: Fatigue; PHQ5: Appetite Disturbance; PHQ6: Feelings of Worthlessness; PHQ7: Concentration Difficulties; PHQ8: Psychomotor Agitation/Retardation; PHQ9: Suicide Ideation; GAD1: Nervous; GAD2: Uncontrollable Worry; GAD3: Worry about Many Things; GAD4: Unable to Relax; GAD5: Restlessness; GAD6: Easily Annoyed; GAD7: Fear of Awful Events.

**Supplemental Figure 3.** Estimation of node centrality difference by bootstrapped difference test.

*Note.* Gray boxes indicate nodes that do not significantly differ from one another, while black boxes represent nodes that do differ significantly (α = 0.05). White boxes show the values of node strength.

SI: Social Isolation; LO: loneliness; PHQ1: Anhedonia; PHQ2: Depressed Mood; PHQ3: Sleep Disturbance; PHQ4: Fatigue; PHQ5: Appetite Disturbance; PHQ6: Feelings of Worthlessness; PHQ7: Concentration Difficulties; PHQ8: Psychomotor Agitation/Retardation; PHQ9: Suicide Ideation; GAD1: Nervous; GAD2: Uncontrollable Worry; GAD3: Worry about Many Things; GAD4: Unable to Relax; GAD5: Restlessness; GAD6: Easily Annoyed; GAD7: Fear of Awful Events.

**Supplemental Figure 4.** Estimation of edge weights difference by bootstrapped difference test.

*Note.* Gray boxes indicate edges that do not significantly differ from one another, while black boxes represent edges with significant difference from one another (α = 0.05). Blue and orange boxes on the diagonal correspond to edge-weights with positive and negative correlations, respectively.
